# Decreased MUC1 in endometrium is an independent receptivity marker in recurrent implantation failure during implantation window

**DOI:** 10.1186/s12958-018-0379-1

**Published:** 2018-06-21

**Authors:** Fangrong Wu, Xiaoyan Chen, Yingyu Liu, Bo Liang, Hui Xu, Tin Chiu Li, Chi Chiu Wang

**Affiliations:** 1 Department of Obstetrics and Gynaecology, The Chinese University of Hong Kong, Shatin, Hong Kong; 2 Li Ka Shing Institute of Health Sciences, Faculty of Medicine, The Chinese University of Hong Kong, Shatin, Hong Kong; 3 School of Biomedical Sciences, Faculty of Medicine, The Chinese University of Hong Kong, Shatin, Hong Kong

**Keywords:** Implantation, Endometrium, Receptivity markers, Reproductive failure

## Abstract

**Background:**

It is postulated that women suffered from recurrent implantation failure (RIF) have different endometrial receptivity compared to those who experienced with idiopathic recurrent miscarriage (RM). In this study, expression of common endometrial markers Leukemia inhibitor factor (LIF), mucin 1 (MUC1) and integrin β3 were studied and compared.

**Methods:**

Fourteen women with RIF, 25 with RM and 20 fertile controls were recruited for endometrial biopsy during implantation window on day LH +  7. Spatial and temporal expression of MUC1, LIF and Integrin β3 were compared using semi-quantitative immunohistochemistry. Association of MUC1, LIF and integrin β3 expression levels with demographic and clinical characteristics were determined.

**Results:**

MUC1 expression in both luminal and glandular epithelium in women with RIF were significantly lower than that in women with RM and fertile controls. There were no differences in LIF and Integrin β3 expression in endometrial epithelium among three groups. Decreased MUC1 expression were not significantly associated with age, BMI, gravidity, parity, cycle length, progesterone level and previous miscarriage.

**Conclusions:**

Deceased expression of MUC1 is an independent marker for endometrial receptivity in RIF women, suggesting MUC1 may contribute to the reproductive failure in RIF women.

**Electronic supplementary material:**

The online version of this article (10.1186/s12958-018-0379-1) contains supplementary material, which is available to authorized users.

## Introduction

Endometrium is critical for a successful implantation [1]. For only a short period of time during mid-luteal phase, the endometrium becomes receptive to the embryo to implant. This specific period has been referred as implantation window around 7 days after surge of luteinizing hormone (LH +  7) [2]. During this implantation window, endometrium will equip with adhesion ligands but remove inhibitory factors to facilitate the implantation process [3]. Many molecules have been proposed as markers for endometrial receptivity, but there is as yet no consensus on which marker is the best. Most of previous studies only focused on a single marker; often the endometrial specimens were not precisely timed; few studies compared the RIF and RM with fertile control at the same time. These may be the potential reasons of the contrasting observation. In addition, the effects of various confounding factors on the result were not examined as well.

Some endometrial receptivity markers expressed in epithelium and others expressed in stroma of the endometrium. Mucin 1(MUC1) is a member-associated protein, highly expressed in luminal and glandular epithelium on LH + 7 day [4, 5]. Fertile women showed a higher level of endometrium MUC1 expression than infertile patients [6]. LIF belongs to interleukin-6 family and has complex regulatory roles in implantation [7]. Animal study showed that blastocysts from LIF knock-out mice failed to implant, however, they were viable when transferred to wild-type recipients with normal endometrium [8]. Integrin is a family of transmembrane glycoprotein that regulates cell-cell and cell-matrix interaction. Integrin isoform β3 expression in endometrium coexists with the period of implantation window [9]. Women underwent IVF treatment with normal Integrin β3 in endometrium had twice pregnancy rate than women with low Integrin β3 level [10]. Abnormal endometrial receptivity may contribute to the reproductive failure. It is postulated that different aspects of endometrial receptivity are disrupted in recurrent implantation failure (RIF) compared with idiopathic recurrent miscarriage (RM). In this study, we studied the expression of three endometrial receptivity markers, MUC1, LIF and Integrin β3 in the same endometrium specimens precisely collected at LH + 7 day and compared among women with RIF, RM and fertile control.

## Materials and methods

### Participants and endometrial biopsy

This study was approved by the ethics committee of The Chinese University of Hong Kong, and informed consent has been obtained from all the participants. Inclusion criteria included women less than 40 years old, with regular menstrual cycle and normal body mass index (BMI), and had no use of hormonal contraception or intrauterine devices for at least 3 months preceding the study. Exclusion criteria included endometrial or uterine pathology such as adenomyosis, fibroids, endometrial polyps and hyperplasia, endometriosis, endometritis, as well as anovulation and polycystic ovary syndrome (PCOS).

RIF was defined as failure to achieve a clinical pregnancy after at least 4 good-quality embryos have been transferred in 3 or more transfer cycles [11]. RM was defined as 3 or more consecutive pregnancy losses before 20-week gestation. All patients were idiopathic with normal uterine cavity examined by ultrasonography and hysterosalpingogram, normal thyroid function, tested negative for lupus anticoagulant and anticardiolipin antibodies, with normal parental karyotype results. Women who had at least one previous live birth within 1–2 years and no history of infertility, implantation failure and miscarriage were included as control.

All participants underwent daily urine test from day 9 of the cycle onwards to identify the LH surge. Endometrial biopsy was obtained using a Pipelle sampler (Prodimed) or Pipet Curet (Cooper Surgical) precisely at LH + 7 day. The endometrial specimens were washed immediately in phosphate-buffered saline (PBS, pH = 7.4) and divided into two parts. One part was fixed in 10% neutral buffered formalin for immunohistochemistry, the other part was sent to pathology examination for endometrial dating by qualified gynecological pathologist blinded to the clinical diagnosis. If the endometrial dating results were not coincident with the endometrium at mid-secretary phase and have been diagnosed as chronic endometritis, the samples were excluded from the study. Chronic endometritis was defined by presence and diagnostic criteria of CD138 plasma cells as described before [12].

### Immunohistochemistry

After overnight formalin fixation and a serial ethanol dehydration, the endometrial specimens were embedded into paraffin wax and sectioned to a thickness of 4 μm. Spatial expression of MUC1, LIF and Integrin β3 in endometrial specimens were determined by standard immunohistochemistry. In brief, sections were dewaxed in xylene, rehydrated through descending ethanol to PBS, and then quenched in 3% hydrogen peroxide in methanol for 20 min. Antigen retrieval was performed in microwave oven with 10 mmol/L sodium citrate buffer (pH = 6.0). Sections were then washed in PBS and blocked in 1% BSA (bovine serum albumin) buffer for 1 h at room temperature, then incubated at 4 °C overnight with primary antibody (goat polyclonal anti-human LIF antibody (1:20, R&D system, AF-250-NA), mouse monoclonal anti-human MUC1 antibody (1:50, abcam, ab8949), or rabbit polyclonal anti-human β3 antibody, (1:50, abcam, ab197662). Then the sections were washed in 0.5% PBST and incubated in appropriate secondary antibody for 1 h at room temperature. The specific antibody binding was visualized by peroxidase substrate 3,3′-diaminobenzidene tetrahydrochloride (DAB, Dako) and counterstained with hematoxylin. Sections were then dehydrated and mounted in synthetic resin DPX. The expression of MUC1, LIF and Integrin β3 in luminal and glandular epithelium were examined under light microscopy (Leica, Germany). Five visual fields were selected under magnification of 400 for analysis. A qualified field was defined as endometrial tissue occupied ≥90% area with both luminal and glandular epithelium.

### H-score analysis

The intensity of LIF, MUC1 and Integrin β3 expression in the endometrial sections were quantified according to the equation: H-score = ∑P_i_, where i was referred as staining intensity (0 = negative; 1 = weak; 2 = moderate; 3 = strong) and P_i_ was referred as percentage of cells stained at each intensity (0–100%). H-score of MUC1, LIF and Integrin β3 in luminal epithelium and glandular epithelium were obtained in 5 qualified 400× visual fields per sample, respectively. Each section was scored independently by two observers both were blinded to the clinical diagnosis. In the event of differences in the score obtained, slides were reexamined until the H-score for the section agreed by both observers. The final scores were averaged from 5 fields for each sample.

### Statistical analysis

Data were analyzed with SPSS 19.0 (IBM, USA). Quantitative data were compared by Mann-Whitney test, and qualitative data were compared by Chi’s square test. H-sores of LIF, MUC1 and Integrin β3 were compared by one-way ANOVA, and then post-hoc LSD test for the multiple-pairwise comparisons. Multivariate linear regression was used to test the association between the receptive markers. A value of *P* < 0.05 was considered to be significant.

## Results

A total of 78 participants were recruited in this study. Of the 78 endometrial specimens, 8 were excluded due to the histological dating not consistent with mid-secretary phase, and another 11 were excluded due to diagnosed of chronic endometritis and history of endometriosis. Immunohistochemistry study was performed on the remained 59 endometrial specimens, including 20 from fertile controls, 14 from RIF women and 25 from RM. Demographic details are summarized in Table 1. Age, number of previous pregnancy, number of live birth and previous miscarriage were significantly different among three study groups, but not BMI and menstrual cycle length. Women in RIF and RM group were older than control fertile women (both *P* < 0.001). However, there was no significant correlation between age and expression of LIF, MUC1 and integrin β3 in both luminal and glandular epithelium (Additional file 1: Table S1).

**Table 1:** Demographic characteristics of participants

Variables	Control (*N* = 20)	RIF (*N* = 14)	RM (*N* = 25)	*P*-value^a^
Age (y)	28.9 ± 3.0	35.2 ± 3.3	36.1 ± 3.2	< 0.001
BMI (kg/m^2^)	21.7 ± 1.9	21.9 ± 2.4	22.9 ± 3.9	0.43
Smoking (n)	0/20 (0%)	0/14 (0%)	3/25 (12.0%)	0.12^a^
Cycle length (d)	29.4 ± 1.7	29.1 ± 2.0	29.8 ± 4.8	0.89
Abnormal karyotype (n)	0/20 (0%)	0/14 (0%)	0/25 (0%)	NA^a^
Progesterone level (nmol/L)	–	62.4 ± 33.5	47.3 ± 20.9	0.31^b^
No. of previous pregnancy				
0	0/20 (0%)	10/14 (71.4%)	0/25 (0%)	< 0.001
1	12/20 (60.0%)	3/14 (21.4%)	0/25 (0%)	< 0.001
≥ 2	8/20 (40.0%)	1/14 (7.1%)	25/25 (100%)	< 0.001
No. of live birth				
0	0/20 (0%)	13/14 (92.9%)	23/25 (92.0%)	< 0.001
1	14/20 (70.0%)	1/14 (7.1%)	2/25 (8.0%)	< 0.001
≥ 2	6/20 (30.0%)	0/14 (0%)	0/25 (0%)	< 0.001
No. of previous miscarriage				
0	20/20 (100%)	10/14 (71.4%)	0/25 (0%)	< 0.001
1	0/20 (0%)	3/14 (21.4%)	0/25 (0%)	< 0.001
2	0/20 (0%)	1/14 (7.1%)	0/25(0%)	< 0.001
≥ 3	0/20 (0%)	0/14 (0%)	25/25 (100%)	< 0.001

Data are presented as mean ± SD or n/N (%). *RIF*, recurrent implantation failure; *RM*, recurrent miscarriage. ^a^Chi square test compared among 3 groups when indicated; ^b^ Mann-Whitney test compared between RIF and RM groups only; NA: not available

**Fig. 1:**
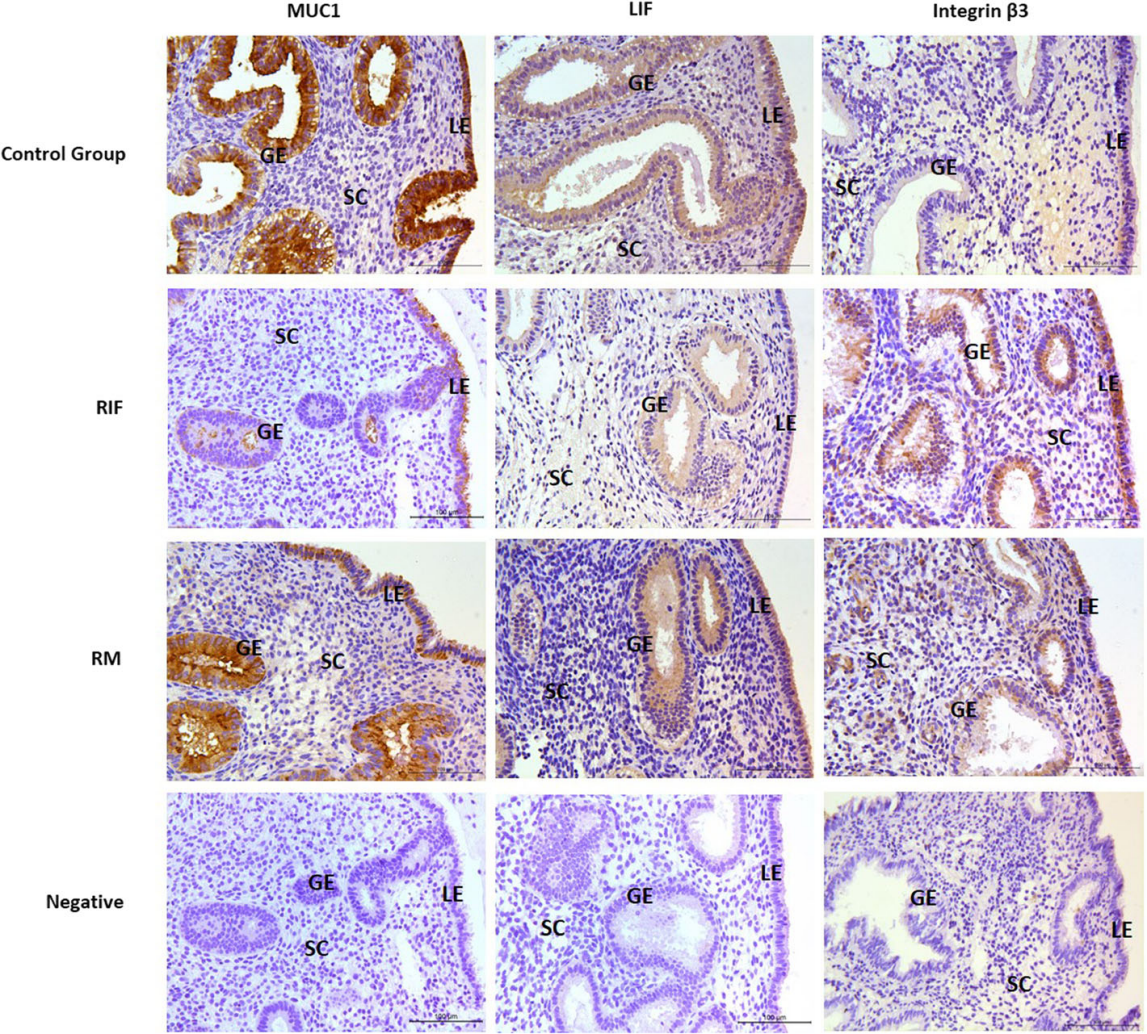
MUC1, LIF and integrin β3 staining in endometrium. *LE*, luminal epithelium, *GE*, glandular epithelium, *SC*, stromal cell. Magnification: X 200. Scale bar: 100μm

MUC1, LIF and Integrin β3 expression were identified in both luminal and glandular epithelium, but LIF and Integrin β3 can also be found in stromal cells (Fig. 1). MUC1 showed strong immunoreactivity in both luminal and glandular epithelial cells in control group, but less intense in RM group and very low in RIF group. LIF showed positive immunoreactivity in the glandular epithelium than that in the luminal epithelium and stromal cells, but overall the immunoreactivity was not as strong as MUC1. Integrin β3 showed positive immunoreactivity in both luminal and glandular epithelium in both RIF and RM groups but less in control group.

**Fig. 2:**
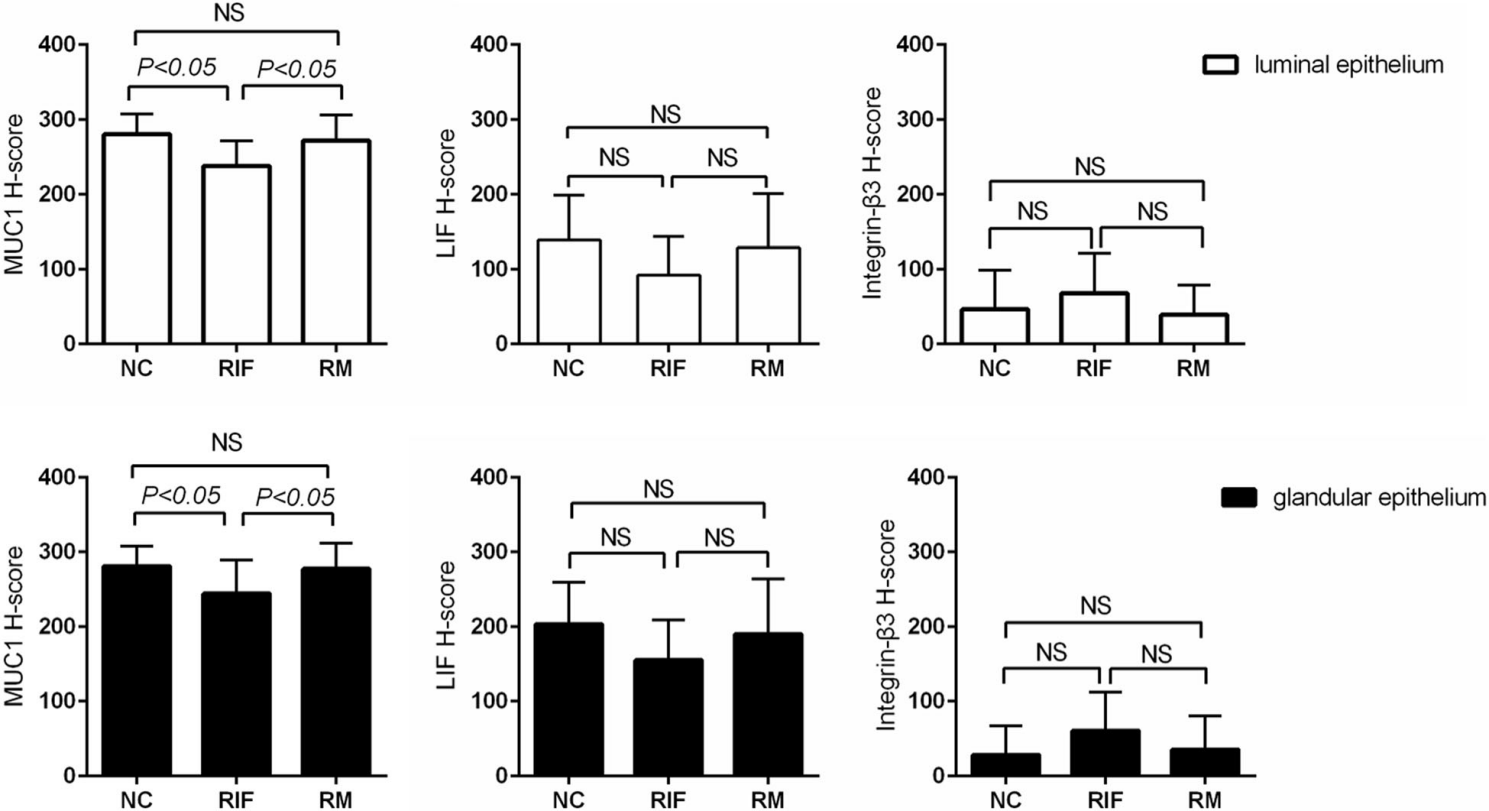
Differentiated expression of MUC1, LIF and integrin β3. H-sores of LIF, MUC1 and integrin β3 expression in luminal (upper panels) and glandular (lower panels) epithelium were compared by one-way ANOVA among groups, and post-hoc LSD test has been used to test the multiple-pairwise comparisons. *NC*, control group; *RIF*, recurrent implantation failure; *RM*, recurrent miscarriage. *NS*, no significant.

The H-score analysis showed that expression of MUC1 in both luminal and glandular epithelium in RIF group was significantly lower than those in control and RM group (Fig. 2). No significant differences in LIF and Integrin β3 expression in either luminal or glandular epithelium among and between the three groups were found, although the expression of LIF in luminal and glandular epithelium in RIF group was slightly lower and expression of Integrin β3 were slightly higher than other groups. Although chronic endometritis and endometriosis were excluded, there were no significant differences in MUC1, LIF and Integrin β3 expressions in the endometrium between women with and women without chronic endometritis and endometriosis (Additional file 1: Table S2).

In multivariate linear regression analysis (Table 2), age, BMI, cycle length, progesterone level, gravidity, parity and previous miscarriage were not significantly associated with endometrial MUC1, LIF and Integrin β3 expression levels. The association remained not significant when the multivariate regression analysis was adjusted by the clinical diagnosis.

**Table 2:** Multivariate linear regression model of demographic and clinical characteristics

Dependent variables	Post-hoc statistical power (*P* < 0.05)	Model summary				Coefficients (constant)		
		R	R^2^	Adjusted R^2^	*P*	B	95%CI	*P*
MUC1 H-score in luminal epithelium	0.99	0.61	0.37	−0.64	0.90	235.52	− 370.43-841.48	0.36
MUC1 H-score in glandular epithelium	0.97	0.57	0.32	−0.77	0.94	226.85	− 402-48-856.19	0.40
LIF H-score in luminal epithelium	0.45	0.34	0.12	−1.29	0.99	257.86	− 1026.67-1542.39	0.63
LIF H-score in glandular epithelium	0.25	0.28	0.07	−1.40	1.00	384.53	− 973.02-1742.07	0.50
Integrin β3 H-score in luminal epithelium	0.99	0.68	0.50	−0.39	0.79	−45.99	− 721.89-629.92	0.87
Integrin β3 H-score in luminal epithelium	0.96	0.55	0.31	−0.81	0.95	54.47	− 745.01-853.95	0.87

Independent variables include age, *BMI*, cycle length, gravidity, parity, number of previous miscarriage and progesterone level on biopsy day. With or without adjustment of clinical conditions: control, RIF and RM; *R*, residual; *R*^*2*^, R square *B*, beta coefficient *SE*, standard error. 95% *CI*, 95% confidence interval for B

## Discussion

Recurrent implantation failure and idiopathic recurrent miscarriage present two major challenges of reproductive failure to clinicians providing care for patients who wish to start or extend their family [13, 14]. The luminal epithelium is the first point of contact between the endometrium and blastocyst, which acts as both barrier and receptor at the same time. In the present study, expression of endometrial receptivity markers LIF, MUC1 and Integrin β3 in luminal and glandular epithelium during the implantation window in women with RIF and RM were examined and compared.

MUC1, LIF and Integrin β3 have long been proposed as biomarkers for endometrial receptivity. Many studies have been carried out; however, the results were inconsistent (Table 3). A special strength of our study is the precise timing of the specimens; all specimens were obtained precisely 7 days after the LH surge. It is particularly important as endometrial morphology and function change rapidly in the peri-implantation period, so that a difference of only one or two days could have introduced significant variance to the results. It also explains why there was significant controversy regarding the observations reported in the literature. One reason for these discrepancies is the different time point of collecting endometrium biopsy for examination, especially if the biopsy specimens were obtained over a period of several days.

**Table 3:** Summary of previous inconsistent studies of MUC1, LIF and integrin β3 expression in endometrium

Source		Study (n)	Fertile control (n)	Timing of biopsy	Methods	MUC1	LIF	Integrin αvβ3/β3
RPL/RM	Xu et al. 2011	30	26	LH + 7–8 day	Immunohistochemistry	↓	↔	↔
	Banerjee et al. 2012	36	30	LH + 5–10 day	Immunohistochemistry Flow cytometric analysis	↓	↓	↓
	Germeyer et al. 2014	21	29	LH + 5–7 day	Immunohistochemistry	NA	NA	↓
	Karaer et al. 2014	30	30	LH + 6–11 day	RT-PCR	NA	↑	NA
	WU et al.2018 ^a^	25	20	LH+ 7 day	Immunohistochemistry	↔	↔	↔
RIF	Mariee et al. 2012	45	15	LH + 7–9 day	Immunohistochemistry	NA	↓	NA
	Coughlan et al. 2013	45	6	LH + 7–9 day	Immunohistochemistry	NA	NA	↔
	Bastu et al. 2015	26	23	LH + 7–9 day	ELISA & Western-blot	↓	NA	NA
	Comba et al. 2015	21	20	LH + 6–10 day	ELISA	NA	↓	NA
	WU et al. 2018 ^a^	14	20	LH + 7 day	Immunohistochemistry	↓	↔	↔

*RPL*, recurrent pregnancy loss *RM*, recurrent miscarriage *RIF*, recurrent implantation failure *RT-PCR*, quantitative real-time *PCR*, *ELISA*, enzyme-linked immunosorbent assay. ↓ expression significantly reduced; ↔ expression no significant difference; ↑ expression significantly increased when compared with control. *NA*, not available; ^a^ present study

MUC1 has been proposed as an anti-adhesive protein because of its physiochemical hindrance mediated by its long ectodomain, which may inhibit the attachment between blastocyst and endometrium. Animal studies have showed that MUC1 is down-regulated before implantation [15, 16], but human studies found that MUC1 is up-regulated during implantation window [4]. Although the precise role of MUC1 in implantation is still unclear, many studies showed low level of MUC1 was associated with impaired receptivity of endometrium [6, 17–19]. Women with RM had reduced endometrial MUC1 expression when compared to fertile women on days LH + 7 and 8 [17]. They proposed that decreased expression of MUC1 may induce endometrial super-fertility and interrupt embryo selection, which allows defective blastocysts to implant but leads to increase miscarriage rate. However, our data did not show significantly decrease of MUC1 in RM group, suggesting MUC1 may not be a reliable receptivity marker for RM. In contrast, MUC1 was significantly decreased in RIF group and multivariate linear regression analysis showed MUC1 was independent of demographic and clinical characteristics of the subjects, regardless of RM, RIF or control. It suggests that decreased endometrium MUC1 expression is an independent receptivity marker in RIF during implantation window.

As for Integrin β3 in endometrium, we found no significant difference among three study groups. Many investigators like us also failed to find significant change in Integrin β3 expression between fertile and RM women [17, 20, 21], although Germeyer et al. observe that women with unexplained pregnancy loss had significantly reduced Integrin β3 expression compared with health control [22]. Coughlan et al. demonstrated that RIF was not associated with abnormal endometrial Integrin expression, and the expression of Integrins α1, α4, and αvβ3 have no prognostic value in subsequent IVF treatment [20]. In this study, we also did not find any significant difference in Integrin β3 among RM, RIF and fertile control and nor any significant correlation between MUC1, LIF and Integrin β3.

In agreement with others, we observed a stronger endometrium staining of LIF in epithelial cells compared with stromal cells. Comba et al. found that both blood and tissue levels of LIF were statistically lower in patients with RPL [23]. Decreased LIF expression level has been demonstrated in RIF patients by other study, when endometrium biopsy was obtained on days LH + 7 to LH + 9 [24]. However, the results of our study showed no significant difference in expression of LIF among three study groups in both luminal and glandular epithelium, even though H-scores showed a tendency of reduced expression in RIF patients.

There are some limitations of our study. First, the total number of participants included in the study is relatively small. The relative small sample size in each of the three groups studies may result small or subtle differences. Nevertheless, the demonstration of significant reduction of MUC1 expression in this suggests that the difference is likely to be of significant biological relevance. Our current sample size allowed us to show a difference of mean H score at least 30 with SD 15 with Power 99% and Type I error of 0.01. Second, the use of immunohistochemistry to examine the expression of various markers is rather semi-quantitative but it has the advantage of obtaining information of spatial expression of the protein markers in different tissue compartments. In our study, we were able to show that the expression of MUC1 in women with RIF was significantly reduced in the both luminal and glandular epithelium. The use of alternative methods such as quantitative real-time polymerase chain reaction or Western blot will not be able to demonstrate the expression of the protein markers in specific cellular components. Thirdly, although MUC1, LIF and Integrin β3 had been separately investigated in different studies, we studied 3 markers simultaneously in the serial sections of same sample; the endometrial specimens were collected precisely during implantation window; and RIF and RM were compared with fertile control in the same study.

## Conclusions

In summary, our study showed MUC1, but not LIF and Integrin β3, was significantly decreased in both luminal and glandular epithelium in women with RIF, but not in women with RM. Both LIF and Integrin β3 do not appear to be sensitive receptivity markers for RIF or RM. In addition, the reduction in MUC1 expression was an independent marker of endometrial receptivity in women with RIF. It suggests MUC1 contributes to the unexplained reproductive failure in RIF. Further in-depth functional and interventional studies of MUC1 in RIF are needed.

## Additional file

Additional file 1: **Table S1.** Relationship between female age and receptivity markers. **Table S2.** H-score comparison between endometritis/endometriosis and non-endometritis/non-endometriosis women. (DOCX 16 kb)
