# Supplementary material for: Decreased MUC1 in endometrium is an independent receptivity marker in recurrent implantation failure during implantation window
Source: Reprod Biol Endocrinol. 2018 Jun 21;16:60. doi: 10.1186/s12958-018-0379-1 (PMC6013892; doi:10.1186/s12958-018-0379-1)
Supplement: Supplementary file 1 — Table S1. Relationship between female age and receptivity markers. Table S2. H-score comparison between endometritis/endometriosis and non-endometritis/non-endometriosis women. (DOCX 16 kb) [file 12958_2018_379_MOESM1_ESM.docx]

**Table S1 Relationship between age and receptivity markers**.

| Markers | age | |
| --- | --- | --- |
|  | Correlation coefficient | P |
| MUC1 in luminal epithelium | -0.22 | 0.11 |
| MUC1 in glandular epithelium | -0.10 | 0.46 |
| LIF in luminal epithelium | -0.09 | 0.55 |
| LIF in glandular epithelium | -0.08 | 0.60 |
| Integrin β3 in luminal epithelium | 0.06 | 0.68 |
| Integrin β3 in glandular epithelium | 0.13 | 0.35 |

Statistical Method: Pearson correlation

**Table S2 H-score comparison between endometritis/endometriosis and non-endometritis/non-endometriosis women**

| Markers | Endometritis and endometriosis group  (n=11) | Non-endometritis and non-endometriosis group  (n=59) | p |
| --- | --- | --- | --- |
| MUC1 in luminal epithelium | 267.0±35.4 | 267.2±43.2 | 0.99 |
| MUC1 in glandular epithelium | 270.5±37.5 | 277.2±27.2 | 0.57 |
| LIF in luminal epithelium | 124.8±65.0 | 119.6±66.3 | 0.81 |
| LIF in glandular epithelium | 187.4±65.8 | 187.6±70.9 | 0.99 |
| Integrin β3 in luminal epithelium | 48.5±48.5 | 49.9±38.4 | 0.93 |
| Integrin β3 in glandular epithelium | 38.9±45.7 | 54.4±56.4 | 0.32 |

Statistical method: Mann Whitney U test
